# KRAS Mutation in Rare Tumors: A Landscape Analysis of 3453 Chinese Patients

**DOI:** 10.3389/fmolb.2022.831382

**Published:** 2022-03-11

**Authors:** Shuhang Wang, Qin Li, Peiwen Ma, Yuan Fang, Yue Yu, Ning Jiang, Huilei Miao, Qiyu Tang, Yuqi Yang, Shujun Xing, Rongrong Chen, Xin Yi, Ning Li

**Affiliations:** ^1^ Clinical Cancer Centre, National Cancer Centre/National Clinical Research Centre for Cancer/Cancer Hospital, Chinese Academy of Medical Sciences and Peking Union Medical College, Beijing, China; ^2^ Geneplus-Beijing Institute, Beijing, China; ^3^ NHC Key Laboratory of Pulmonary Immunological Diseases, Guizhou Provincial People’s Hospital, Guiyang, China

**Keywords:** rare tumors, KRAS, G12C, targeted therapy, immunotherapy

## Abstract

KRAS is the most commonly mutated oncogene in human cancers. Targeted therapy and immunotherapy for this gene have made remarkable progress in recent years. However, comprehensive molecular landscape analysis of KRAS in rare tumors is lacking. Retrospective analysis was performed on clinical samples from patients with rare tumors collected between September 2015 and September 2021, using hybrid-capture-based next-generation sequencing for genomic profiling and immunohistochemistry assay for PD-L1. Of the 3,453 patients included in analysis, KRAS mutations were identified in 8.7% patients in overall; mutation rate and mutation subtypes varied widely across tumor systems and subtypes. KRAS mutations included 21 missense mutations, of which G12D (29.2%), G12V (24.6%), and G13D (10.8%) were most common. Interestingly, KRAS G12C was observed in 0.6% patients overall, and in 5.7% of sarcomatoid carcinoma of the lung and 5.4% of clear cell ovarian cancer tumors, but none in small-bowel cancer tumors. 31.8% KRAS mutations and 36.4% KRAS G12C mutations co-occurred with other targetable alterations. No significant correlation was observed between TMB-H, MSI-H, PD-L1 status, and KRAS mutation status, which may be related to the high proportion of G12D. This study is the first KRAS mutation landscape study in rare tumors of large sample size in China and worldwide. Our results suggest that targeted therapy and immunotherapy are both feasible, albeit complex, in these patients. This information may have significant impact on the operation of clinical trials for rare tumor patients with KRAS mutations in China.

## Introduction

Rare cancers are roughly defined as cancers with fewer than 15 new diagnoses in 100,000 people a year, but there is no universally adopted definition (Abbas-Aghababazadeh et al., 2020). For example, sarcomatoid carcinoma of the lung (SARCL) is rare tumor, with <3% of nonsmall-cell lung cancer (NSCLC) (Fallet et al., 2015). Collectively rare cancer account for more than 20% of all cancer diagnoses in a year (Abbas-Aghababazadeh et al., 2020). Clear cell ovarian cancer, CCOV are rare aggressive, chemo-resistant tumors comprising approximately 13% of all epithelial ovarian cancers, which have distinct clinical and molecular features, when compared to other gynecological malignancies (Khalique et al., 2020). Neuroendocrine tumors (NETs) are a group of rare neoplasms originating from dispersed neuroendocrine cells, mainly of the digestive and respiratory tract (Poblocki et al., 2020). Gastrointestinal stromal tumors (GIST) are the most common of the rare non-epithelial neoplasms of gastrointestinal tract, accounting for 0.1–3% of gastrointestinal malignancies (Al-Share et al., 2021).

Kirsten rat sarcoma (KRAS) gene is the most commonly mutated oncogene in human cancers. KRAS mutations are identified in approximately 90% of pancreatic cancers, 30–40% of colon cancers, and 15–20% of lung cancers, mostly non-small-cell lung cancer (NSCLC) (Pylayeva-Gupta et al., 2011; Singh et al., 2015). Apart from genetic alterations associated with spontaneous tumor development, it has been increasingly appreciated that KRAS mutation also exhibits a broad impact on the tumor microenvironment, which helps to promote and maintain the malignancy of cancer (Young et al., 2013; Kortlever et al., 2017; Ambrogio et al., 2018; Dias Carvalho et al., 2019). In addition to the exciting progress in the development and approval of targeted therapeutic drugs for KRAS (Hallin et al., 2020; Hong et al., 2020; Jänne et al., 2020), many studies have been carried out investigating the effects of KRAS mutations on immunotherapy (Kim et al., 2017; Lee et al., 2018; Köhler and Jänne, 2021).

Our team has previously studied the genomic profiling of rare tumors in China, which indicated that employing targeted therapy and immunotherapy in rare tumors is highly feasible (Wang et al., 2020; Wang et al., 2021). However, the molecular landscape of KRAS mutation in rare tumors has not been studied. This retrospective study describes the landscape of KRAS mutation in 3,453 Chinese patients with rare tumors, in order to provide valuable information for the operation of clinical trials for patients with rare tumors which harbor KRAS mutations in China.

## Methods

### Patient Recruitment

According to the definition and update of rare tumors established by the China National Cancer Centre (Wang et al., 2020), we collected and retrospectively analysed genomic profiling data of 3,453 patients with rare tumors from the Geneplus database. This database contained patients enrolled from multiple hospitals in China from September 2015 to September 2021. All patients received hybrid-capture-based next-generation sequencing (NGS) testing in Geneplus-Beijing Institute after obtaining written informed consent. Meanwhile, all the patients were stratified into different clinicopathological subgroups according to OncoTree system (http://oncotree.mskcc.org/). Tumor subtypes with more than 30 samples were also included in the analysis. The final data analysed included 3,453 cases with NGS data, 1,697 cases of tumour mutational burden (TMB), 1,436 cases of microsatellite instability (MSI) status and 488 cases of programmed cell death ligand-1 (PD-L1) expression.

### Next-Generation Sequencing

All samples were tested by NGS in the Geneplus-Beijing laboratory, which is accredited by American College of Pathologists (Sun et al., 2019; Wang et al., 2020; Zhuo et al., 2020; Wang et al., 2021). Briefly, all tissue samples were hematoxylin-eosin stained and reviewed to ensure a minimum of 20% tumor cells. Tumor DNA and ctDNA were extracted from serial sections of formalin fixed paraffin embedded (FFPE) tumor tissue and 4–5 ml plasma, respectively. Genomic profiling was performed on hybridization-captured, adaptor ligation–based libraries to a minimal mean effective depth of coverage of 100× in leukocytes, 300× in tumor tissue and 1,000× in cell-free DNA samples, for 59 or 1,021 cancer-related genes. All classes of genomic alterations were identified, including single nucleotide variants (SNV), small insertions and deletions (InDels), copy number alterations, and rearrangements.

TMB was defined as the number of somatic nonsynonymous SNV and InDels per megabase of the coding region, with allele frequency ≥0.03 in tumor tissue sample or ≥0.005 in ctDNA sample respective. The threshold for high tumour mutational burden (TMB-H) was identified as the top quartile and determined to be greater than 9 mutations per megabase (Jia et al., 2018; Wang et al., 2019). microsatellite instability high (MSI-H) was defined by MSIsensor score greater than 8 (Niu et al., 2014).

### PD-L1 Expression

PD-L1 expression was assessed in FFPE tumor tissues using the PD-L1 immunohistochemistry (IHC) 22C3 pharmDx kit and Dako Automated Link 48 platform (Agilent Technologies, Santa Clara, CA, United States) in 414 patients and using the SP263 pharmDx kit and Ventana Bench-Mark XT automated staining platform (Ventana Automated Systems, Inc., Tucson, AZ, United States) in 74 patients. The methodology for PD-L1 tests was performed according to standard protocols. Positive PD-L1 expression was identified using a cut-off of greater than 1%.

### Statistics

The Chi-square test or Fisher’s exact test was performed to analyse the correlation between KRAS and immunotherapeutic molecular markers. All statistical analysis was performed with SPSS (v.23.0; STATA, College Station, TX, United States) or GraphPad Prism (v. 6.0; GraphPad Software, La Jolla, CA, United States) software. Statistical significance was defined as a two-sided *p*-value of <0.05.

## Result

### Clinicopathological Characteristics of Patients

Three thousand four hundred and fifty-three patients (3,453) with rare tumors were included in this study. Table 1 summarizes the clinicopathological characteristics of all patients. The median age was 56, and male patients accounted for 55.1% (1901/3,453). Among these patients, tumor tissue samples were available for genetic analysis for 2,595 patients. Cell-free tumor DNA (ctDNA) was available for 842 patients, pleural effusion samples for 17, peritoneal effusion samples for 14, and cerebrospinal fluid (CSF) samples for 3. These 3,453 cases included 122 rare tumor subtypes, with soft tissue (1,076 cases), digestive (9,310), neural (732), cancer of unknown primary (CUP) (262), and respiratory (207) systems as the top 5 affected bodily systems. 33.4% of patients underwent surgery and 32.0% of patients received systemic therapy. The other 34.6% had no available treatment records.

**TABLE 1:** Clinicopathological characteristics of patients.

Characteristic	Pts. (*N* = 3,453) (%)
Age, years	
median	56
range	1–97
Gender	
female	1,520 (44.0%)
male	1901 (55.1%)
unknown	32 (0.9%)
Specimen	
tumour tissue	2,595 (75.2%)
ctDNA	824 (23.9%)
pleural effusion	17 (0.5%)
peritoneal effusion	14 (0.4%)
CSF	3 (0.1%)
System	
1.head and neck	52 (1.5%)
2.digestive	931 (27.0%)
3.respiratory	207 (6.0%)
4.reproductive	81 (2.3%)
5.urinary	12 (0.2%)
6.multiple system	57 (1.7%)
7.skin	24 (0.7%)
8.soft tissue	1,076 (31.2%)
9.bone	11 (0.3%)
10.endocrine	8 (0.2%)
11.neural	732 (21.2%)
12.CUP	262 (7.6%)
Treatment	
operation	1,152 (33.4%)
systemic therapy	1,106 (32.0%)
not recorded	1,195 (34.6%)

### Genomic Profiling of KRAS

Somatic mutations were detected in 94.03% of patient samples (3,247/3,453), with TP53 (31.9%), KIT (16.4%), TERT (12.9%), CDKN2A (12.8%), and EGFR (11.1%) as the top 5 mutant genes. The top 20 mutant genes are summarized in Supplementary Table S1). 11.9% (202/1,697) of samples were TMB-H, 1.5% (22/1,436) were MSI-H and 45/3% (221/488) were PD-L1 positive.

**FIGURE 1:**
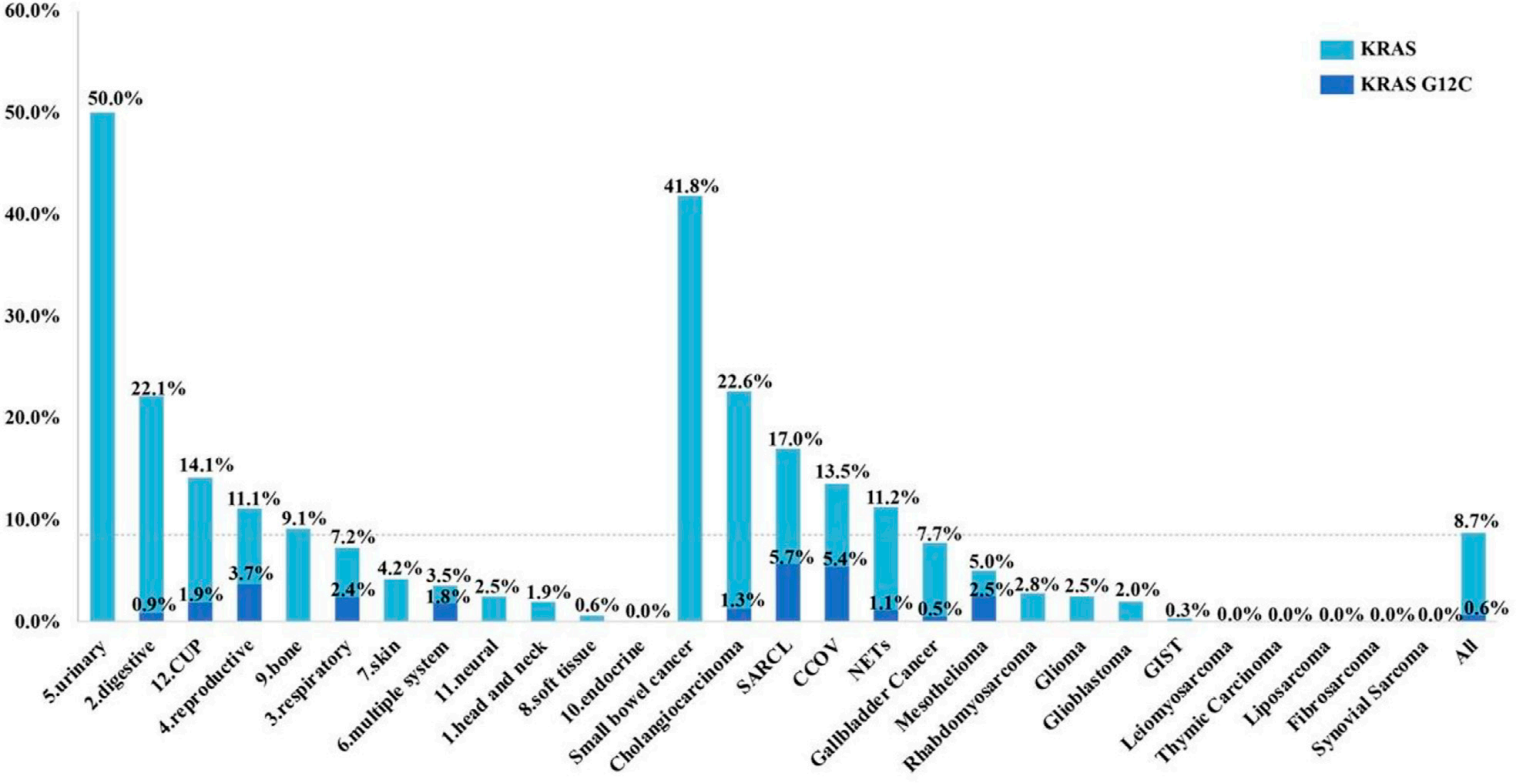
Prevalence of KRAS mutations and KRAS G12C in different systems and subtypes of rare tumors. Cancer of unknown primary, CUP; sarcomatoid carcinoma of the lung, SARCL; clear cell ovarian cancer, CCOV; neuroendocrine tumors, NETs; gastrointestinal stromal tumor, GIST.

KRAS mutations were identified in 302 patient samples (8.7%). Prevalence of KRAS mutation varied widely across tumor systems, ranging from 0% in patients with endocrine system tumors to 50.0% in patients in urinary system disease. The five systems with the highest rate of KRAS mutation were urinary (50.0%, 6/12), digestive (22.1%, 206/931), CUP (14.1%, 37/262), reproductive (11.1%, 9/81), and bone (9.1%, 1/11). With respect to tumor subtypes, small bowel cancer (41.8%, 51/122), cholangiocarcinoma (22.6%, 125/553), sarcomatoid carcinoma of the lung (SARCL) (17.0%, 9/53), clear cell ovarian cancer (CCOV) (13.5%, 5/37), and neuroendocrine tumors (NETs) (11.2%, 20/178) had the highest rates of KRAS mutation, while the mutation rates in glioma and glioblastoma were only about 2%. KRAS mutations were extremely rare in a variety of sarcoma subtypes (Figure 1 and Supplementary Table S2).

**FIGURE 2:**
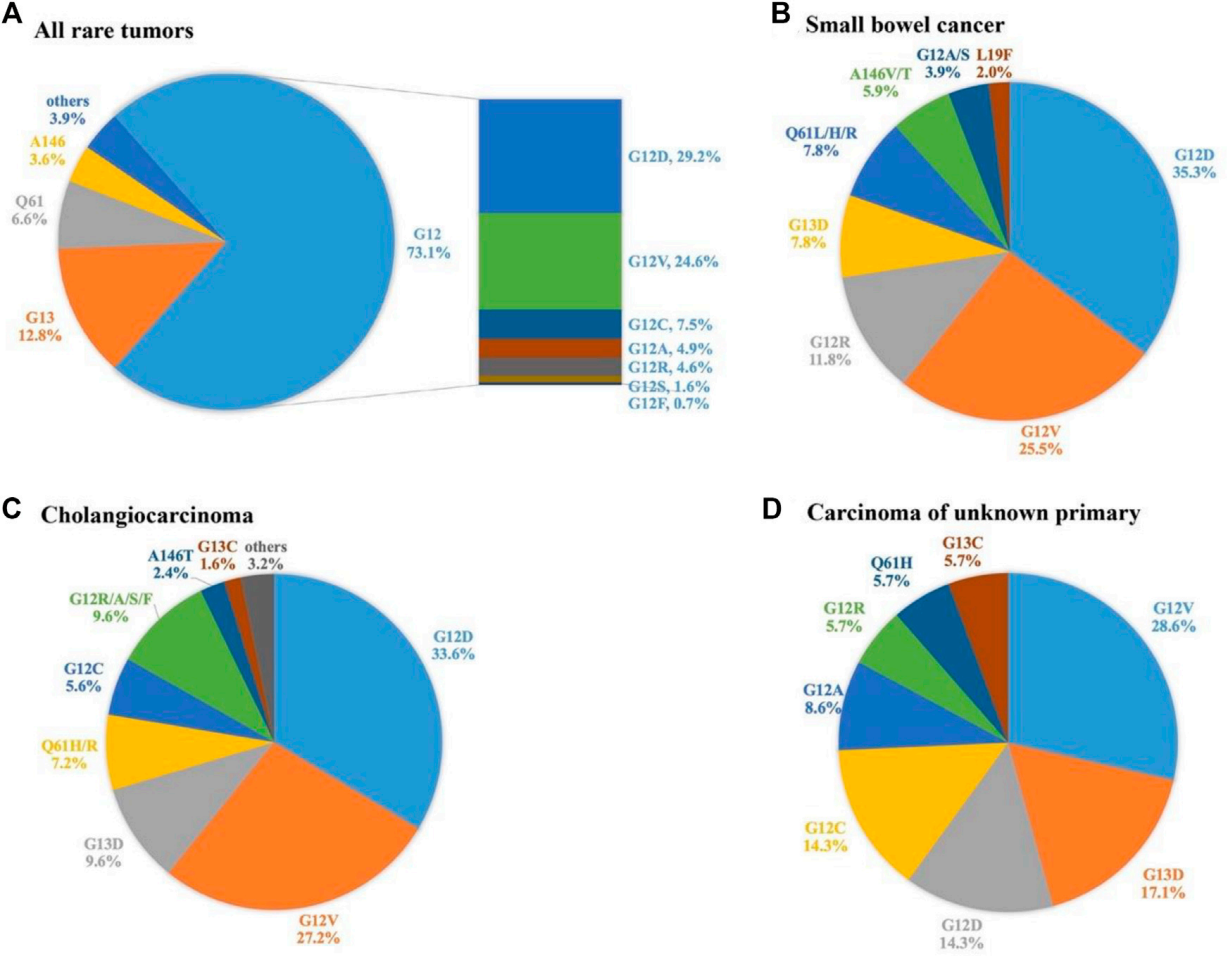
Distribution of KRAS mutations in all rare tumors and three subtypes.

A total of 305 KRAS mutations were detected, including 21 missense mutations (Table 2). The most common mutations were G12D (29.2%), G12V (24.6%), and G13D (10.8%). Overall, mutations were mainly concentrated in G12 (72.5%, 223/305), followed by G13 (13.1%, 39/305) and H61 (6.9%, 20/305). The 3.6% (11/305) mutation rate of T146 mutation is also noteworthy. We further analysed the distribution of KRAS mutation subtypes in 3 tumor subtypes with high KRAS mutation rate and sufficient sample size, namely small bowel cancer, cholangiocarcinoma, and CUP. G12D, and G12V were the main mutations in small bowel cancer, and G12C mutation was not detected. KRAS mutations in cholangiocarcinoma are heterogeneous, mainly in G12D and G12V mutations, and G12C was observed in 5.6% of patients. In contrast, the mutation subtypes of CUP were relatively simple. The incidence of G12V, G13D, G12D, and G12C were 28.6, 17.1, 14.3 and 14.3%, respectively, (Figure 2).

**TABLE 2:** KRAS mutations identified in rare tumors.

Mutation	Mutated No.	Mutated rate (%)
G12D	89	29.2
G12V	75	24.6
G13D	33	10.8
G12C	23	7.5
G12A	15	4.9
Q61H	15	4.9
G12R	14	4.6
A146T	8	2.6
G13C	6	2.0
G12S	5	1.6
Q61L	5	1.6
Q22K	3	1.0
A146V	3	1.0
G12F	2	0.7
Q61R	2	0.7
E63K	2	0.7
L19F	1	0.3
D33E	1	0.3
A59G	1	0.3
T74P	1	0.3
K117R	1	0.3
Total	305	100.0

### Correlation of KRAS With Other Genes and Molecular Markers

97% of KRAS mutated tumors have other gene alterations. 140 targetable alterations co-mutated with KRAS were identified in 102 patients (33.8%), of which *CDKN2A, PIK3CA* and *ATM* were the top three co-mutated genes, while *ALK*, *NTRK1/2/3*, *RET* and *ROS1* were not observed to be co-mutated with *KRAS* in any patients. Notably, 36.4% of *KRAS* G12C patients also had targetable alterations (Table 3).

**TABLE 3:** Targetable alterations co-mutated with KRAS.

Gene	Targetable genomic alteration	Co-mutated case No.	Co-mutated rate (%)	Co-mutated with G12C case No.	Co-mutated rate (%)
*CDKN2A*	loss, substitution, truncation	36	11.9	3	13.6
*PIK3CA*	substitution, amplification	19	6.3	3	13.6
*ATM*	substitution, truncation	18	6.0	1	4.5
*IDH1,2*	substitution	12	4.0		
*NF1*	loss, truncation	10	3.3		
*PTEN*	loss, substitution, truncation	10	3.3	1	4.5
*BRCA2*	substitution, truncation	8	2.6		
*ERBB2*	amplification, substitution	7	2.3		
*BRAF*	substitution, fusion	5	1.7		
*FGFR1,2,3*	substitution, amplification, fusion	5	1.7		
*MET*	amplification	5	1.7		
*BRCA1*	substitution, truncation	2	0.7		
*KIT*	substitution	2	0.7		
*EGFR*	substitution	1	0.3		

**FIGURE 3:**
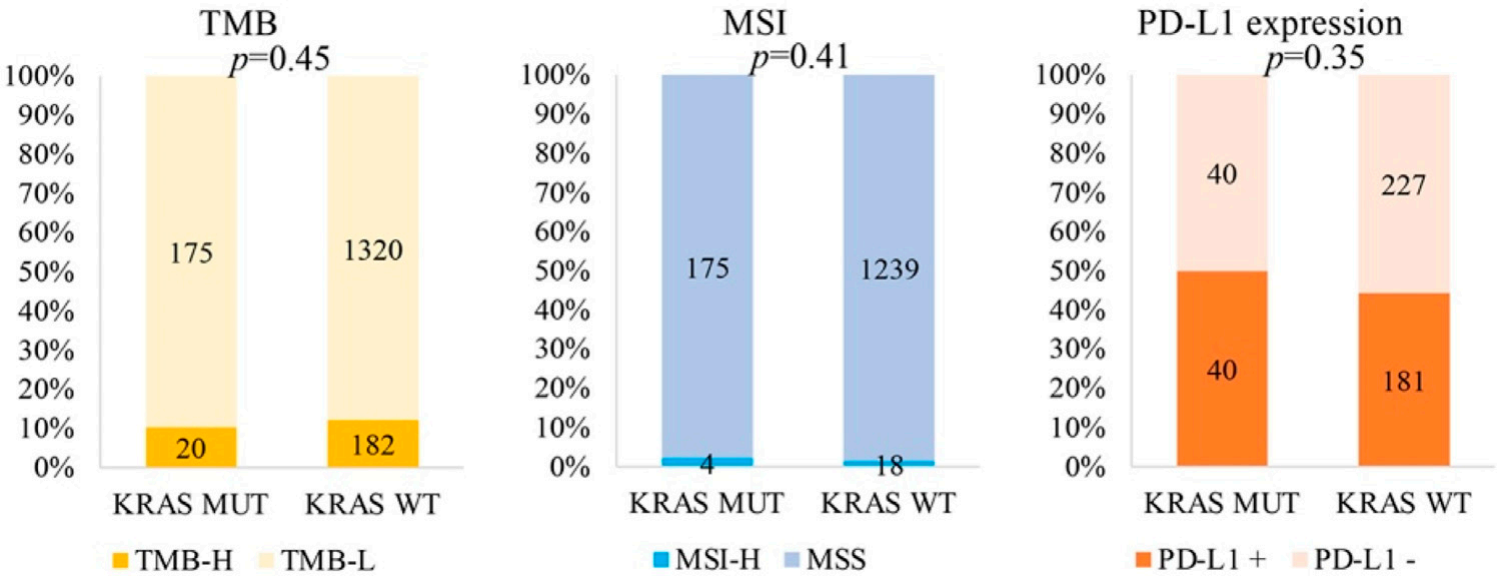
Correlation of KRAS with immunotherapeutic molecular markers.

By analysing the correlation between *KRAS* and immunotherapeutic molecular markers, we found that *KRAS* mutation was not related to TMB-H (*p* = 0.45), MSI-H (*p* = 0.41) or PD-L1 (*p* = 0.35) (Figure 3).

## Discussion

This study reveals the KRAS mutation landscape of rare tumors in China for the first time. To our knowledge, it is also the first study with such a large sample size of rare tumors worldwide. This study provides valuable information with regard to the feasibility of targeted therapy and immunotherapy in KRAS-mutated rare tumors.

*KRAS* is the most frequently mutated oncogene in human cancers, with mutations in about 30% of all cancers, although the prevalence varies greatly in different tumors (Uprety and Adjei, 2020). In this study, KRAS mutations were identified in 8.7% rare tumors. Importantly, not only mutation rate, but also mutation subtypes varied widely across tumor systems and subtypes. We identified some interesting commonalities. The 41.8% KRAS mutation rate and prevalence of G12D/V mutations in small cancer was similar to that in colorectal cancer (Moore et al., 2020). In addition to small bowel cancer and cholangiocarcinoma, we found a high prevalence of KRAS mutations in SARCL, OOCV, CUP, and NETs. The prognostic and predictive value of KRAS in these rare tumor subtypes deserves further study.

In recent years, the development and approval of *KRAS* G12C inhibitors has attracted extensive attention (Hallin et al., 2020; Hong et al., 2020; Jänne et al., 2020). The mutation rate of *KRAS* G12C in cholangiocarcinoma and CUP was similar to that in previous study (Nassar et al., 2021), suggesting that it is feasible to select patients with these tumor subtypes for clinical trials. However, in this study, although the *KRAS* mutation rate in small bowel cancer reached 41.8%, there was no G12C mutation detected, which was different from the G12C mutation rate of 3.1% in previous study (Nassar et al., 2021). This study observed that the proportion of KRAS G12C mutations in colorectal cancer and non-small cell lung cancer samples was higher in whites than in Asians. Interestingly, 5.7% of SARCL patients and 5.4% of CCOV patient samples were observed to have KRAS G12C in this study, indicating that such patients should also be considered in clinical trials of KRAS G12C inhibitors.

We found that 33.8% KRAS mutations co-occurred with other known targetable alterations (36.4% in KRAS G12C), which was similar to rates of co-mutation observed in NSCLC and CRC (Loong et al., 2020). This substantial frequency of co-mutation leads to high complexity of targeted treatment strategies in a considerable number of patients, when considering whether to use KRAS G12C inhibitors or other approved drugs with known targetable alterations. Combination therapy may be a feasible strategy.

The predictive and prognostic value of KRAS mutation status in immunotherapy of solid tumors remains controversial (Kim et al., 2017; Jeanson et al., 2019). Studies have shown that KRAS mutation in NSCLC is significantly correlated with PD-L1 positive (Schoenfeld et al., 2020), and KRAS/TP53 co-mutation in lung adenocarcinoma is associated with higher TMB (Xiang et al., 2020). In this study, it was found that KRAS was not associated with TMB-H, MSI-H, or PD-L1 positivity in rare tumors. This may be due to the different KRAS mutation subtypes present in this study. In the analysis of different KRAS mutation subtypes in lung adenocarcinoma, it was found that the most common mutant subtype of KRAS was G12C, which was associated with higher TMB and PD-L1 positivity rate compared with wild type KRAS. This study also found that the proportion of patients with G12D was much lower, TMB was lower than that of wild-type tumors, and PD-L1 positivity in patients with TP53 co-mutation was significantly lower (Gao et al., 2020). In our study, the proportion of KRAS mutation G12D was as high as 24%, which may be the reason why KRAS mutation has no significant correlation with TMB-H and PD-L1 positivity.

Previously, the investigation of KRAS mutations in rare tumors is still limited. In this study, we analyzed the KRAS mutation landscape in rare tumors of large sample size in China. A previous study identified 27.2% of KRAS mutations in SARCL of France using mass spectrometry (Fallet et al., 2015). In this study, they reveal that KRAS mutations were always found to involve codon 12, with KRAS G12C being the most frequently found (63.6%). Most KRAS mutations (72.7%) were found in tumors with adenocarcinoma component. In our analysis, we found a 17% KRAS mutations in SARCL. The most common mutations were G12D (29.2%), G12V (24.6%), and G13D (10.8%). Another analysis indicates a 14% KRAS mutations at codon12, exon 2, of KRAS in CCOV of Italy, in which G12V, G12A, and G12S are found (Zannoni et al., 2014). In our analysis, we found a 13.5% KRAS mutations in CCOV. These data identify the difference of KRAS mutation rate and types of rare tumors between China and Europe. This might have a different role of G12C KRAS inhibitors in this latter population. Moreover, compared with KRAS, HER-2 amplification, BRAF mutation and NTRK fusion may have more clinical significance for targeted therapy even if the incidence of those gene alterations is low. The mutation landscape of HER-2, BRAF, and NTRK should be investigated in the system in future analysis.

There are many therapeutic strategies with KRAS as molecular marker. In addition to targeting therapy and immune checkpoint inhibitors, adoptive T-cell therapy has also been studied. Adoptive T-cell therapy targeting mutant KRAS G12D in colon cancer might mediate effective antitumor immunotherapy (Tran et al., 2016; Sim et al., 2020). In view of the high proportion of KRAS G12D mutation of 29.2% in rare tumors, the application of adaptive T-cell therapy in rare tumors is also worth pursuing.

## Conclusion

Through this first KRAS mutation landscape study of large sample size rare tumors in China and worldwide, we have found that KRAS mutation is common in rare tumors, with an overall mutation rate of 8.7% and a G12C mutation rate of 0.6%, with mutation rate and mutation subtypes varying widely across tumor systems and subtypes. Interestingly, 5.7% of SARCL tumors and 5.4% of CCOV tumors were observed to harbour KRAS G12C mutations. As 33.8% of KRAS patients have other targetable alterations, and KRAS mutation has no correlation with TMB-H, MSI-H, and PD-L1 positivity, it suggests that targeted therapy and immunotherapy are feasible but may be complex in patients with rare tumors with KRAS mutation. This information may have significant impact on operation of clinical trials in rare tumor patients with KRAS mutation in China.

## Data Availability

The original contributions presented in the study are included in the article/Supplementary Material, further inquiries can be directed to the corresponding authors.

## Supplementary Material

Supplementary Table S1 Top 20 mutant genes of all rare tumor patients.

Supplementary Table S2 Prevalence of KRAS mutations and KRAS G12C in different systems and subtypes of rare tumors.

## Abbreviations

NSCLC, non-small-cell lung cancer; NGS, next-generation sequencing; TMB, tumour mutational burden; MSI, microsatellite instability; PD-L1, programmed cell death ligand-1; FFPE, formalin fixed paraffin embedded; IHC, immunohistochemistry; CUP, cancer of unknown primary; SARCL, sarcomatoid carcinoma of the lung; CCOV, clear cell ovarian cancer; NETs, neuroendocrine tumors.
